# Dual Careers of Athletes During COVID-19 Lockdown

**DOI:** 10.3389/fpsyg.2021.657671

**Published:** 2021-04-01

**Authors:** Pascal Izzicupo, Angela Di Baldassarre, Ilvis Abelkalns, Ugis Bisenieks, Antonio Sánchez-Pato, Francisco José Cánovas-Alvarez, Mojca Doupona, António J. Figueiredo, Juan Alfonso García-Roca, Barbara Ghinassi, Alejandro Leiva-Arcas, Lourdes Meroño, Anda Paegle, Liliana-Elisabeta Radu, Cristian-Mihail Rus, Oana-Mihaela Rusu, Hugo Sarmento, Janis Stonis, Raquel Vaquero-Cristóbal, Vasco Vaz, Laura Capranica

**Affiliations:** ^1^Department of Medicine and Aging Sciences, University “G. D'Annunzio” of Chieti-Pescara, Chieti, Italy; ^2^University of Latvia, Riga, Latvia; ^3^Areté Research Group, Faculty of Sport, Universidad Católica San Antonio de Murcia, Murcia, Spain; ^4^Department of Sport Sociology and History, Faculty of Sports, University of Ljubljana, Ljubljana, Slovenia; ^5^European Athlete as Student Network, Ghaxaq, Malta; ^6^Research Unit for Sport and Physical Activity, Faculty of Sport Sciences and Physical Education, University of Coimbra, Coimbra, Portugal; ^7^Faculty of Physical Education and Sport, University “Alexandru Ioan Cuza”, Iaşi, Romania; ^8^Injury Prevention in Sport Research Group, Faculty of Sport, Universidad Católica San Antonio de Murcia, Murcia, Spain; ^9^Department of Movement, Human and Health Sciences, University of Rome Foro Italico, Rome, Italy

**Keywords:** dual-career athletes, student-athlete, COVID-19, lockdown, active lifestyle, academic commitment, sport commitment

## Abstract

This study aimed to investigate the student-athletes' capability to face the academic, sport, and social challenges during the coronavirus disease 2019 (COVID-19) lockdown and to disclose novel aspects of dual careers. A 32-item online survey encompassing demographic characteristics, sport and university engagement, support and dual-career benefits, physical activity, sitting time, and the time deemed necessary to recover the previous level of performance was developed. Four hundred sixty-seven student-athletes (males: 57%, females: 43%) from 11 countries, competing in 49 different sports (individual: 63.4%, team: 36.6%) at regional (17.5%), national (43.3%), and international (39.2%) levels, and enrolled at high school (21.9%) and university (78.1%) levels completed the survey. During the lockdown, the respondents decreased the time dedicated to sport and academics, although they maintained an active lifestyle. Student-athletes from countries under severe contagion were more likely to train at home, dedicate to academics, and receive support from the coach but less likely receive support from their teachers. With respect to their team sport counterparts, athletes competing in individual sports trained more and were more likely to receive support from their coaches. International athletes showed the highest training time and support from their coaches and as student-athletes. High school students received more support from their coaches and teachers, whereas university students were more likely considering dual careers useful to cope with the COVID-19 pandemic. This study substantiates the relevant role of competitive sports participation in the maintenance of active lifestyles, with student-athletes considering home training and e-learning valuable resources during the lockdown. Furthermore, their sport and academic commitments helped student-athletes cope with the emergency of the COVID-19 pandemic.

## Introduction

In late December 2019, a cluster of patients with pneumonia of unknown etiology was linked to a seafood wholesale market in Wuhan, China. The outbreak of coronavirus disease 2019 (COVID-19) was declared to be a public health emergency of international concern on January 30, 2020, and finally recognized as a pandemic on March 11, 2020. The human-to-human transmission was considered a major transmission mode, with isolation deemed the most effective means of containing COVID-19 (Wang et al., 2020). Thus, many countries established a lockdown, albeit with varying degrees of restrictions, which determined unprecedented disruption of lives and work at all population levels (Zhang et al., 2020).

Although experts indicated youth as less susceptible to the virus compared to older people, several countries adopted the closure of schools, colleges, universities, and other educational institutions to break the chain of transmission. These measures have affected >80% of the world's student population (UNESCO, 2020), with recommendations encompassing online scheduled curriculum-based study, basics of hygiene, maintenance of daily routine, need for indoor physical activity, and sedentary behavior reduction (Ghosh et al., 2020; Ricci et al., 2020). Actually, educational institutions not fully accustomed to online lessons have rapidly moved from face-to-face to online delivery mode of teaching and distance evaluation, trying to overcome the problems due to possible unavailability of technological support and lack of computer skills of teachers and students. The effort of the whole university community and the institutions provided a responsible, prompt, and agile response in order to guarantee the continuation of academic activities by adapting to remote methods (Torrecillas, 2020). Although this passage was not easy, it may be considered an important opportunity to invest in teaching technologies that can represent a vital resource for those students who frequently cannot attend classes, such as hospitalized students and elite athletes (Abenza-Cano et al., 2020).

In light of a possible increased risk of respiratory tract viral infections associated with high-intensity physical activity with long durations (Halabchi et al., 2020), on March 2020, national governments and international sporting committees implemented COVID-19-related measures suspending or canceling sport participation and events (Parnell et al., 2020). Such a decision had a dramatic impact on active lifestyles, well-being, and quality of life of youth (Cluver et al., 2020), who were the most active European citizens regularly exercising or playing sports (European Commission, 2018). The in-home isolation period severely affected the physical capabilities and psychological well-being of talented and elite athletes who did not train in appropriate sport-related environments, with the proper equipment, and under the supervision of multidisciplinary support teams. In fact, the lockdown and the postponement of major 2020 sport events might have affected the athletes' levels of perceived stress and dysfunctional psychobiosocial states, as well as the aspirations and self-fulfillment of many elite athletes who might have perceived a sport career disruption (di Fronso et al., 2020; Samuel et al., 2020; Taku and Arai, 2020). Despite that athletes might expect changes during their competitive career (e.g., injuries, a transition between competition levels, modifications in sport regulations) and might foresee an end of their sport career, the combination of a higher education and a sport career (e.g., dual careers) is strongly envisaged to ensure a holistic development of the sportspersons and to help them manage the range of changes they are potentially experiencing (European Commission, 2012; Stambulova et al., 2020).

Educated athletes are crucial investments for the development of the future society, especially for the skills they developed in managing challenges at educational and sport levels, such as life management, communication skills, emotional awareness, and career planning (European Commission, 2012; B-WISER, 2018; Wylleman and De Brandt, 2019). In this framework, the impact of COVID-19 is particularly relevant for student-athletes, who present a “centaur” profile (Pato, 2017) and a relevant commitment to academic and sport (Condello et al., 2019), supported by their high motivation to pursue dual careers across two domains of central importance to their lives (Gaston-Gayles, 2005; Guidotti et al., 2014; Lupo et al., 2015). In the literature, they have been considered resilient against adversity and burnout, determined in maintaining dual-career perspectives and capable of benefiting from a mechanism for life skills transfer (Comeaux and Harrison, 2011; Aquilina, 2013; Sorkkila et al., 2017; Barger and Seward, 2018; Stambulova et al., 2020). In 2013, Aquilina identified eight reasons student-athletes might consider in making their decision to pursue dual careers, including the development of life skills useful in different contests, such as putting things in perspective, maintaining interest and commitment, and a “sense of balance.” Barger and Seward (2018) claimed that a merged student-athlete identity could serve as a mechanism for life skills transfer, especially motivation. However, athletes' motivation and engagement in the social and academic domains are also dependent on a dual-career culture, positive entourage, support services, and different eligibility criteria of talented and elite athletes eligible for dual-career paths and programs (Comeaux and Harrison, 2011; Amsterdam University of Applied Sciences, 2016; Capranica and Guidotti, 2016; Stambulova and Wylleman, 2019).

Notwithstanding, in most European countries, dual-career policies are not fully implemented, with Member States addressing this issue with their own approaches, generally categorized as (Aquilina and Henry, 2010): (1) State-centric regulation, in which government legislation or statutory regulations place responsibilities on higher education institutions to provide flexible academic paths; (2) State as a sponsor/facilitator, in which states promote formal agreements to meet athletes' needs at the educational level; (3) National Sporting Federation/Institute as an intermediary, in which national governing or sports bodies negotiate flexible academic paths with educational institutions; and (4) Laisser-faire/No Formal Structures, in which individually negotiated agreements are arranged, when possible. In the latter case, sports and education are considered distinct and separate, often impairing the holistic development of student-athletes and interfering or disrupting favorable exchanges of experiences between careers (Barger and Seward, 2018). Despite the recommendation of European Guidelines on Dual Career of Athletes to establish structured dual-career policies and services (European Commission, 2012), many European athletes need to negotiate their dual-career paths with academic and sport staff, often having no support for distance learning and flexible schedules.

In considering the unexpected challenges of COVID-19, information on the student-athletes' capability to face the academic, sport, and social challenges (e.g., long-distance relationships, forced coexistence, parents and sport clubs' economic difficulties) during this specific situation is unique and could contribute to disclose novel aspects of dual careers. So far, there are no other known circumstances in recent times in which athletes around the world have had most of their competitions and training canceled. These circumstances could condition the psychological state of student-athletes, affecting not only their sporting but also their student side, as there is a great transfer of skills and events from one side to the other (Comeaux and Harrison, 2011; Aquilina, 2013; Sorkkila et al., 2017; Barger and Seward, 2018; Stambulova et al., 2020).

Therefore, the main purpose of the present study was to investigate the effects of the COVID-19 pandemic lockdown on: (i) the student-athletes' sport and university engagement before and during the COVID-19 lockdown; (ii) the support received at sport and academic levels, if any; (iii) the student-athletes' perceptions of possible dual-career benefit during the lockdown; and (iv) the athletes' active lifestyles and time needed to resume their sporting performance. It was hypothesized that different effects of the COVID-19 pandemic on student-athletes' sport and academic engagement, support, active lifestyle, and perception of personal skills to cope with the emergency would emerge in relation to the severity of the contagion in the countries of enrolled student-athletes, the academic level, the competitive sports level, and the sport typology.

## Materials and Methods

The institutional review board of the University of Rome Foro Italico approved this study (CAR46/2020), carried out in agreement with the Declaration of Helsinki. Due to a lack of a clear definition of talented and elite athletes adopted at the European level (Capranica and Guidotti, 2016), in the present study, student-athletes were considered those recognized by their respective sports organizations as a member/candidate of national team, regardless of the league and the age category in which they compete. In considering that dual-career policies, services, and support vary between European Member States, the study included three countries with formal dual-career policies and agreements (e.g., State-centric regulations: Portugal and Spain; State as sponsor/facilitator: Latvia) and three countries with no formal structures (e.g., Laisser-faire: Italy, Romania, and Slovenia).

### The Instrument

At present, no specific instruments to collect relevant information on student-athletes during exceptional events such as the COVID-19 pandemic are available. Thus, the conceptualization of the factors that could contribute to uncovering how student-athletes cope with the unexpected COVID-19 pandemic challenges was achieved by means of the eminence-based data preliminarily gathered with elite student-athletes from six Member States (i.e., Italy, Latvia, Portugal, Romania, Slovenia, and Spain) participating in the European ERASMUS+ project More Than Gold (603346-EPP-1-2018-1-LV-SPO-SCP), the FISU-EAS questionnaire for student-athletes (Condello et al., 2019), and the International Physical Activity Questionnaire (IPAQ, De Courten, 2002; Lee et al., 2011). In considering that short questionnaires have a higher response rate (Deutskens et al., 2004) to gather information on the dual careers of athletes during COVID-19, a 32-item survey consisting of original items was constructed and a Web instrument was selected to reach student-athletes competing for at least six Member States (i.e., Italy, Latvia, Portugal, Romania, Slovenia, and Spain) and to allow multimedia and self-administration (Callegaro et al., 2015). The questionnaire had been validated in a pilot study, following the indications of Carretero-Dios and Pérez (2005), and its validity had been analyzed through a panel of experts, obtaining a concordance index of 0.92, with agreement in all items above 80%. In particular, its reliability was analyzed, with Cronbach's alpha reliability coefficients above 0.75, revealing that the instrument had adequate internal consistency with composite reliability indices above 0.70 and Maximum shared variance (SMV) above 0.50. The questionnaire contained the following blocks of questions (Supplementary File 1):

- sociodemographic characteristics of the student-athletes to frame this population (Q1–8), including the athlete's sex, age, academic level (high school, university major and level-bachelor, master's, and PhD), athletic level (e.g., regional, national, international), practiced sport, country, and national COVID-19 lockdown measures at university and competition levels
- the athletes' engagement in sport and university (Q9–16), encompassing the time devoted to sport and academics before and during the COVID-19 lockdown, and the location of training during the COVID-19 lockdown
- any support the athletes received at sport, academic, and dual-career levels and their typology (Q17–22)
- the student-athletes' perceptions of dual-career benefit during the lockdown (if any) with respect to athletes not enrolled at educational level (Q23–25)
- the time spent in Vigorous-Intensity Physical Activity (VPA, ≥6 METs), Moderate-Intensity Physical Activity (MPA, 3–5.9 METs), and Moderate to Vigorous-Intensity Physical Activity (MVPA, ≥3 METs); sitting time during a typical weekday and a weekend day (Craig et al., 2003) before and after the COVID-19 lockdown; and the time required to resume their sporting performance once the lockdown restrictions are removed (Q26–32).

To collect data on a large sample of a heterogeneous population, close-ended questions were privileged (e.g., single-response checklist type). Student-athletes were allowed to elaborate further on their answers for questions related to their reasons and the modalities of training (or not), the type of support they received (or not), and the reasons why their position as student-athletes helped (or not) them during the COVID-19 lockdown.

### Recruitment

To meet the country-specific regulations of General Data Protection Regulations and privacy rights of personal data, the national Partners engaged in the More Than Gold project were required to recruit the target population of student-athletes through their networks. Participants were informed that their contribution was voluntary and anonymous and that they could withdraw from the study at any time without providing any reason. Thus, student-athletes followed a link to the online survey, and informed consent was assumed from the respondents' compilation. To increase a response cumulative of the COVID-19 lockdown, the survey was launched on May 1, 2020, and was closed on May 15, 2020, with a follow-up contact with 7 days in between. According to the literature (Deutskens et al., 2004), this procedure was deemed necessary to increase the response rate for the online surveys encompassing more than 20 items. However, this procedure did not allow calculation of the probability and response rates, as well as inferential statistics, being a not-list-based survey (Callegaro et al., 2015).

### Data Analysis

Sport typology (Q6) was categorized as individual and team sports. Open-ended questions were read several times until the content was familiar to two independent researchers and allowed for immersion in the research (Miles and Huberman, 1994). Therefore, the researchers independently created a series of original categories for each question and discussed them to reach an agreement. Finally, the researchers individually positioned each response in one or more categories before comparing and satisfying an agreement. When an agreement could not be reached, a third researcher was asked. The answers to the question “How was your training modified?” (Q13) were categorized as very and little modified, endurance and strength training, and sport-specific. The answers to the question “If not [trained]: specify why?” (Q14) were categorized as poor motivation and poor conditions. The answers to the question “What support your coach provided you?” (Q18) were categorized as psychological and moral support, planning, using multimedia devices, video lessons. The answers to the question “What support your teacher provided you?” (Q20) were categorized as psychological and moral support, planning, homework, video lessons, and flexibility. The answers to the question “What support did you receive as a student-athlete?” (Q22) were categorized as psychological and moral support, planning, video lessons, flexibility, tutoring, economic support, and certification/license. The answers to the question “If yes: Why being a student-athlete helped you manage the COVID-19 pandemic better than athletes not enrolled in higher education?” (Q24) were categorized as time management, discipline, motivation, health, and support. The answers to the question “If not: Why being a student-athlete did not help you manage the COVID-19 pandemic better than athletes not enrolled in higher education?” (Q25) were categorized as no difference, commitments, and lack of support.

Physical activity level, the time spent at various physical activity intensities, and sitting time (Q26–31) were obtained according to the guidelines for data processing and analysis of the IPAQ short and long forms (De Courten, 2002; Lee et al., 2011). According to the severity of contagion (cumulative incidence in relation to the population), countries were divided into severe (≥300 cases per 100,000 inhabitants) and light–mild (<300 cases per 100,000 inhabitants) contagion (COVID-19 Map—Johns Hopkins Coronavirus Resource Center, 2020).

Data were analyzed using the Statistical Package for the Social Sciences version 24.0 (SPSS Inc., Chicago, Illinois) and included descriptive statistics of the frequency of occurrence (expressed in absolute values or percentages) for single responses (e.g., Q1–5, Q7–12, Q15–17, Q19, Q21, Q23, Q26–32). For Q9, Q11, and Q15–16 participants' own responses, the frequency of occurrence (expressed in absolute values or percentages) was calculated considering three classes of occurrence for the time engaged in sport and university (≤10, 11–20, ≥21 h week^−1^). For inferential statistics, an *a priori* level of significance was set at *P* < 0.05. First, the Kolmogorov–Smirnov test was applied to ascertain the normality of data distribution for the data from Q1–5, Q7–12, Q15–17, Q19, Q21, Q23, Q26–32. Since data were not normally distributed, Kruskal–Wallis test followed by Mann–Whitney was applied to assess the effects of the independent variable competition level on the dependent variables weekly VPA, weekly MPA, weekly MVPA, weekdays sitting time, weekend days sitting time, and total sitting time. *Post hoc* analysis was applied using Bonferroni adjustment for *P*-value interpretation. To assess the effects on the same dependent variables of the independent variables contagion severity, academic level, and sport typology, the Mann–Whitney test was applied. Thus, chi-square tests were applied to verify the distribution of answers regarding time devoted to sports commitments and training before and after the lockdown, engagement in training and location, time dedicated to academics before and after the lockdown, support from coach and teachers, support as student-athlete, coping with the emergency due to the outbreak of COVID-19 as student-athlete, the physical activity level, and time needed to recover the previous performance level in relation to the contagion severity, academic level, sport, and competition level. *Post-hoc* analysis was applied using the calculation of adjusted residuals with Bonferroni adjustment for *P*-value interpretation. Finally, Wilcoxon signed rank test was applied to investigate the differences between sports and academic engagement before and after the lockdown. *Post-hoc* analysis was applied using Bonferroni adjustment for *P*-value interpretation.

## Results

### Sociodemographic Characteristics of the Student-Athlete Population (Q1–8)

Overall, 477 student-athletes responded to the survey. Empty questionnaires (*n* = 8) and those from respondents who were not student-athletes (*n* = 2) were removed from the dataset. The study included a final sample of 467 student-athletes (age: 21.6 ± 4.7 years; males: 57%, females: 43%) from 11 countries. The majority of the respondents were Italian (55.1%), followed by Romanian (14.0%), Spanish (11.9%), Latvian (6.9%), Portuguese (6.3%), Slovenian (2.4%), Finnish (1.9%), Kazakhstani (0.9%), Croat (0.2%), Serbian (0.2%), and British (0.2%). A higher proportion (73.1%) of athletes were living in countries experiencing a severe contagion (e.g., Italy, Portugal, Spain, and United Kingdom) with respect to that of those (26.9%) living in countries suffering a light–mild contagion (e.g., Romania, Latvia, Slovenia, Finland, Kazakhstan, Croatia, and Serbia).

**Figure 1:**
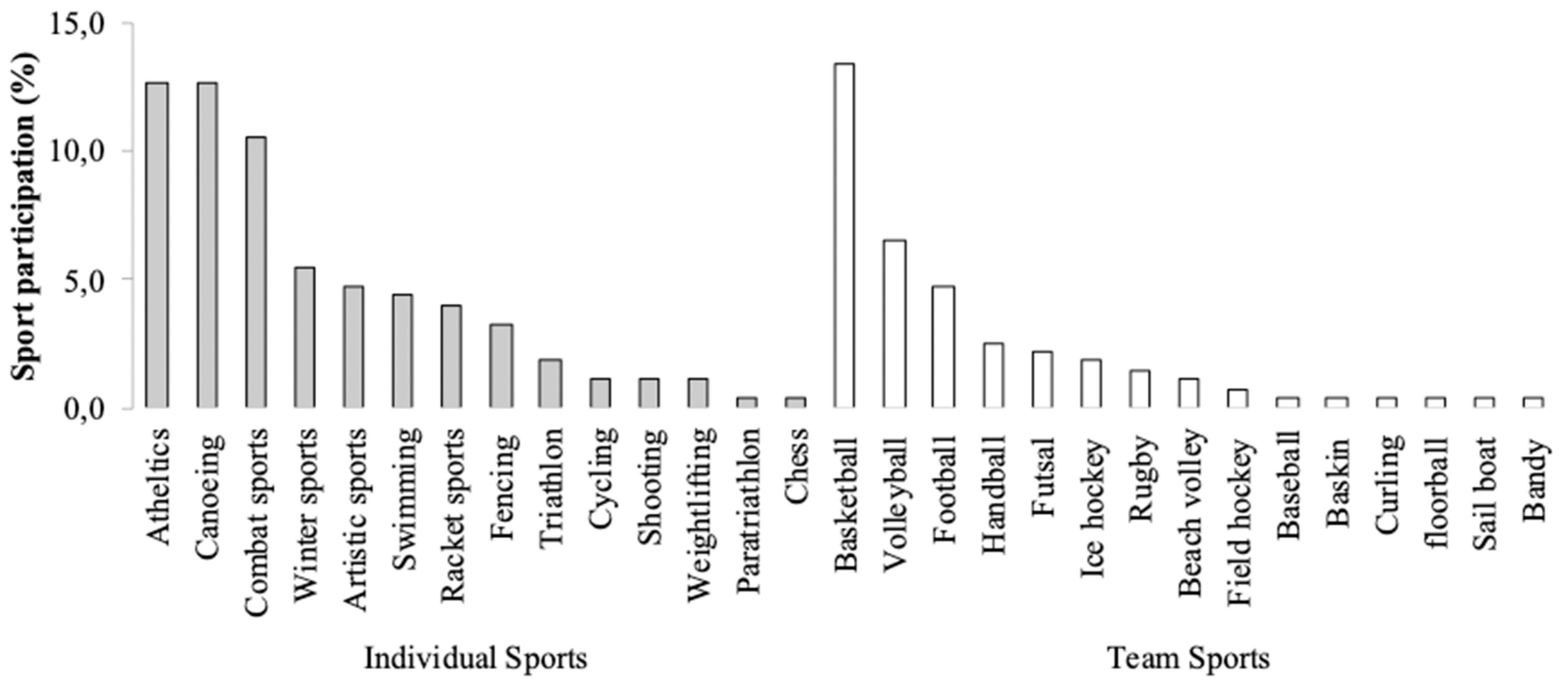
Frequency of occurrence (%) of respondents in relation to the student-athletes sport disciplines and sport typology (e.g., individual and team sports).

The participants competed in 49 different sports, with a higher proportion of individual sports (63.4%) with respect to team (36.6%) ones (Figure 1). Athletes competing at the national level outnumbered (43.3%) their international (39.2%)- and regional (17.5%)-level counterparts. While 21.9% of athletes were enrolled at high school level, those enrolled at university level (78.1%) were mainly bachelor students (77.0%), followed by those pursuing a master's (18.7%) and PhD (4.3%) degrees.

### Student-Athlete's Engagement in Sport and University Before and During the COVID-19 Lockdown (Q9–16)

**Figure 2:**
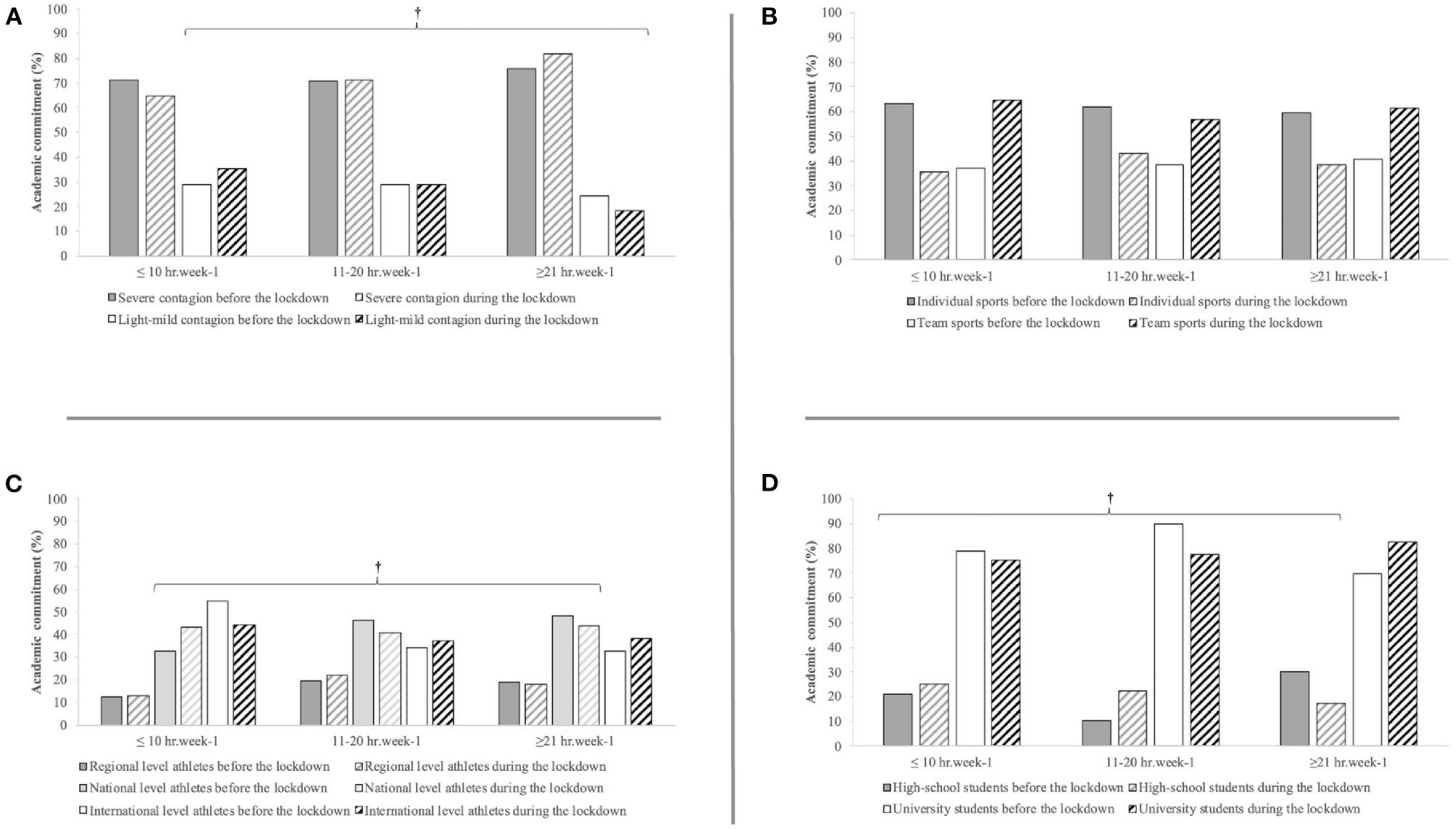
Weekly time of academic commitment of student athletes in relation to the severity of contagion **(A)**, the typology of sport **(B)**, competition level **(C)**, and academic level **(D)**. †Significant decrease for light–mild contagion countries (*P* = 0.002), national level athletes (*P* = 0.002), and high school students (*P* < 0.001).

**Figure 3:**
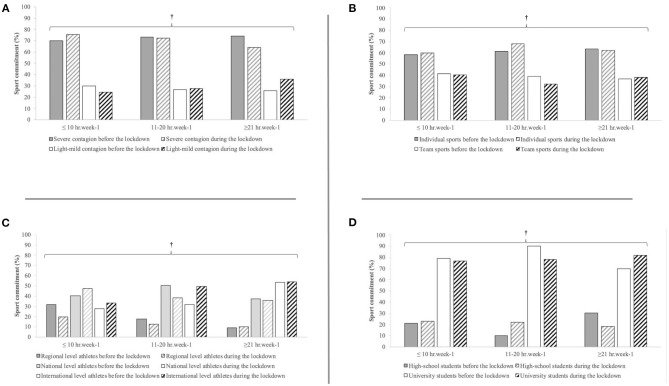
Weekly time of sport commitment of student athletes in relation to the severity of contagion **(A)**, the typology of sport **(B)**, competition level **(C)**, and academic level **(D)**. †Significant decrease across all subpopulations (*P* < 0.001).

The COVID-19 pandemic lockdown negatively affected both the student-athletes' academic (Z = −1.97, *P* = 0.048) (Figure 2) and sport (Z = −13.8, *P* < 0.001) commitments (Figure 3). Regarding the academic commitment, a significant decrease for light–mild contagion countries (Z = −3.14, *P* = 0.002), national-level athletes (Z = −3.14, *P* = 0.002), and high school students (Z = −6.75, *P* < 0.001) was observed. In particular, a decrease in the ≥21 h week^−1^ class and increases in the ≤10 h week^−1^ class were observed for light–mild contagion countries (Figure 2A), a decrease in both the 11–20 h week^−1^ and ≥21 h week^−1^ classes and an increase in the ≤10 h week^−1^ class were observed for national-level athletes (Figure 2C), and a decrease in the ≥21 h week^−1^ class with a concomitant increase in the ≤10 h week^−1^ and 11–20 h week^−1^ classes were observed for high school students (Figure 2D). Despite almost the totality of the respondents declared to train during the COVID-19 lockdown (90.9%) and mainly at home (94.9%), their commitment to sport decreased for the 11–20 and ≥21 h week^−1^ classes, whereas it increased for the ≤10 h week^−1^ class (Figure 3).

Table 1 summarizes the effects of COVID-19 on the independent variables severity of contagion, academic level, sports type, and competition level on studying and training. In relation to the severity of the contagion, main effects emerged only for training at home (χ^2^ = 14.1, *P* < 0.001) and for the time dedicated to academic activities during the lockdown (χ^2^ = 11.4, *P* = 0.003). In particular, during the lockdown, athletes living in countries experiencing a severe contagion showed highest training at home and academic commitments with respect to their counterparts from the other countries. In considering the academic commitment, a significant difference emerged for the time dedicated to study before the outbreak of COVID-19 pandemic (χ^2^ = 18.5, *P* < 0.001). In particular, high school students were more likely dedicating ≥21 h week^−1^ (*z* = 3.7, *P* < 0.001) with respect to university students. Compared to team sports athletes, those competing in individual sports dedicated more time to training during the lockdown (χ^2^ = 7.7, *P* = 0.006).

**Table 1:** Frequency of occurrence (%) of reported student-athletes' sport and academic commitments before and during the lockdown in relation to the severity of contagion, sport typology, competition level, and academic level.

	**Severity of contagion**		**Sport typology**		**Competition level**			**Academic level**	
	**Severe**	**Light–mild**	**Individual**	**Team**	**Regional**	**National**	**International**	**High school**	**University**
**Sport commitment (training** **+** **competition) before the lockdown**									
≤ 10 h week^−1^ (%)	70.0	30.0	58.5	41.5	32	40.2	27.8	21.1	78.9
11–20 h week^−1^ (%)	73.4	26.6	61.1	38.9	17.7	50.5	31.7	10.1	89.9
≥21 h week^−1^ (%)	74.1	25.9	63.5	36.5	9.2	37.4	53.4	30.2	69.8
**Total (%)**	**73.1**	**26.9**	**63.4**	**36.6**	**17.5**	**43.3**	**39.2**	**21.9**	**78.1**
**Training during the lockdown**									
Yes (%)	74.2	25.8	64.4	35.6	16.3	43.2	40.5	21.4	78.6
No (%)	61.9	38.1	37.0	63.0	26.2	45.2	28.6	27.5	72.5
**Total (%)**	**73.1**	**26.9**	**63.4**	**36.6**	**17.5**	**43.3**	**39.2**	**21.9**	**78.1**
**Training time during the lockdown**									
≤ 10 h week^−1^ (%)	75.6	24.4	59.5	40.5	19.7	47.2	33.1	23.1	76.9
11–20 h week^−1^ (%)	72.4	27.6	67.9	32.1	12.7	38.1	49.2	22.1	77.9
≥21 h week^−1^ (%)	64.0	36.0	62.1	37.9	10.0	36.0	54.0	18.4	81.6
**Total (%)**	**73.1**	**26.9**	**63.4**	**36.6**	**17.5**	**43.3**	**39.2**	**21.9**	**78.1**
**Training at home during the lockdown**									
Yes (%)	74.8	25.2	62.5	37.5	17.2	43.2	39.6	20.8	79.2
No (%)	39.1	60.9	50.0	50.0	8.7	52.2	39.1	34.8	65.2
**Total (%)**	**73.1**	**26.9**	**63.4**	**36.6**	**17.5**	**43.3**	**39.2**	**21.9**	**78.1**
**Academic commitment before the lockdown**									
≤ 10 h week^−1^ (%)	71.3	28.7	63.2	36.8	12.6	32.6	54.8	21.1	78.9
11–20 h week^−1^ (%)	70.9	29.1	61.7	38.3	19.3	46.4	34.3	10.1	89.8
≥21 h week^−1^ (%)	75.6	24.4	59.4	40.6	19.2	48.3	32.6	30.2	69.8
**Total (%)**	**73.1**	**26.9**	**63.4**	**36.6**	**17.5**	**43.3**	**39.2**	**21.9**	**78.1**
**Academic commitment during the lockdown**									
≤ 10 h week^−1^ (%)	64.9	35.1	35.6	64.4	12.7	43.3	44.0	25.0	75.0
11–20 h week^−1^ (%)	71.3	28.7	43.2	56.8	22.1	40.7	37.1	22.3	77.7
≥21 h week^−1^ (%)	81.8	18.2	38.6	61.4	17.8	43.9	38.2	17.3	82.7
**Total (%)**	**73.1**	**26.9**	**63.4**	**36.6**	**17.5**	**43.3**	**39.2**	**21.9**	**78.1**

Before the COVID-19 pandemic, sport commitment increased in relation to the competition level. Compared to their counterparts, athletes competing at international level maintained the highest values for the ≥21 h week^−1^ class before (χ^2^ = 37.2, *P* < 0.001) and during (χ^2^ = 14.4, *P* = 0.006) the lockdown. While the international athletes population showed the lowest academic commitment before the lockdown (χ^2^ = 18.3, *P* = 0.001), no difference between subgroups during the pandemic emerged.

With respect to the open questions, only 2.1% of student-athletes declared to have maintained their training regimes, whereas 84.3% of respondents reported training changes, 4.4% did not describe how they changed their training, and 9.1% did not train mainly due to lack of the proper training conditions at home (56%), lack of motivation (32%), and infection (12%). The student-athletes who changed their training during the lockdown reported to have performed mainly conditioning training, such as strength (44.7%) and aerobic (5.9%) or both (49.4%), although 8.1% in addition performed some sport-specific drills alone at home or in the backyard. While the majority (88.1%) of the respondents who continued to train did not provide details regarding mood and training conditions, 1.5% lamented a decreased motivation and 10.4% a lack of adequate equipment or facilities.

### Support the Student-Athletes Received During the COVID-19 Lockdown at Sport, Academic, and Dual-Career Levels (Q17–22)

Table 2 summarizes the effects of COVID-19 on the support the student-athletes received during the COVID-19 lockdown in relation to the independent variables academic level, sport typology, and competition level. The majority of the respondents declared that they have received support from their coaches (75.8%) and teachers (76.8%), whereas only 25.1% reported a dual-career support. In particular, high school students declared the highest support from their coaches (χ^2^ = 16.9, *P* < 0.001) and teachers (χ^2^ = 4.7, *P* = 0.03), whereas respondents from countries with severe contagion declared the highest support from their coaches (χ^2^ = 13.0, *P* < 0.001) and the lowest support from their teachers (χ^2^ = 7.8, *P* = 0.005). Furthermore, individual sports athletes reported the highest support from their coaches (χ^2^ = 12.0, *P* = 0.001). Also, competition level showed an effect on support from the coaches (χ^2^ = 43.1, *P* < 0.001) and dual-career support (χ^2^ = 13.6, *P* < 0.001). In particular, the *post-hoc* analysis revealed that the international-level athletes received the highest dual-career (*z* = 3.5, *P* < 0.001) and their coaches (*z* = 4.3, *P* < 0.001) support, whereas regional-level athletes received the lowest support from their coaches (*z* = 6.2, *P* < 0.001).

**Table 2:** Frequency of occurrence (%) of reported student-athletes' perceived support from sport, academic, and dual-career staff in relation to the severity of contagion, sport typology, competition level, and academic level.

	**Severity of contagion**		**Sport typology**		**Competition level**			**Academic level**	
	**Severe**	**Light–mild**	**Individual**	**Team**	**Regional**	**National**	**International**	**High school**	**University**
**Support from coach**									
Yes (%)	77.7	22.3	67.1	32.9	10.9	44.3	44.9	26.3	73.7
No (%)	60.4	39.6	42.4	57.6	36.4	41.8	21.8	7.5	92.5
**Total (%)**	**73.1**	**26.9**	**63.4**	**36.6**	**17.5**	**43.3**	**39.2**	**21.9**	**78.1**
**Support from teacher**									
Yes (%)	70.0	30.0	38.6	38.6	15.9	41.7	42.3	24.1	75.9
No (%)	83.8	16.2	61.8	38.2	20.2	48.1	31.7	14.0	86.0
**Total (%)**	**73.1**	**26.9**	**63.4**	**36.6**	**17.5**	**43.3**	**39.2**	**21.9**	**78.1**
**Support as student-athlete**									
Yes (%)	67.5	32.5	66.7	33.3	9.6	36.8	53.5	16.2	83.8
No (%)	74.9	25.1	58.8	41.2	19.5	45.5	35	23.5	76.5
**Total (%)**	**73.1**	**26.9**	**63.4**	**36.6**	**17.5**	**43.3**	**39.2**	**21.9**	**78.1**

In general, the coaches provided training plans (50.9%), as well as psychological (15.5%) and moral (12.0%) support, or a combination (training plans + psychological support = 9.9%; training plans + moral support = 6.4%; moral + psychological support = 5.3%). While the majority of the respondents who received support from coaches (69.9%) did not provide details regarding the means used, 13.1% declared they received support through multimedia communication and 17% through videos. At the academic level, the teachers mainly provided educational support (72.5%) through video lessons, electronic material, and homework and planning; moral and psychological support (18,1%); flexibility for the lessons and examinations (4.1%); or a combination of moral/psychological support + educational support (5.0%) and moral/psychological support + flexibility for the lessons and examinations (0.3%).

### Student-Athletes' Perceptions of Dual-Career Benefit During the Lockdown (Q23–25)

When student-athletes were asked to compare themselves with their non-dual-career counterparts, the majority of the respondents (61.6%) perceived their student-athletes status useful to cope with the emergency of the COVID-19 pandemic. They perceived a higher capacity to manage their time (36.9%) and maintain their motivation (24.3%) and discipline (8.7%), even combining two of these characteristics (17%) and better health (2.9%). Furthermore, 8.7% perceived an advantage due to the received support, even in combination with a higher capacity to manage their time (1.0%) and motivation (0.5%). With respect to their high school counterparts, university student-athletes perceived their dual career positive to cope with the COVID-19 pandemic outbreak (χ^2^ = 13.7, *P* < 0.001). On the other hand, 38.4% declared not to perceive a positive effect for being dual-career athletes. In particular, 57.7% of them acknowledged to be not different from other people, 24.1% to have too demanding commitments, and 2.9% lamented the lack of support. The remaining 15.9% was not able to furnish a reason or a complete answer.

### Student-Athletes Activity and Sedentary Behaviors Before and During the COVID-19 Lockdown and Time Necessary to Recover the Sport Performance Before the COVID-19 Pandemic Lockdown (Q26–32)

**Figure 4:**
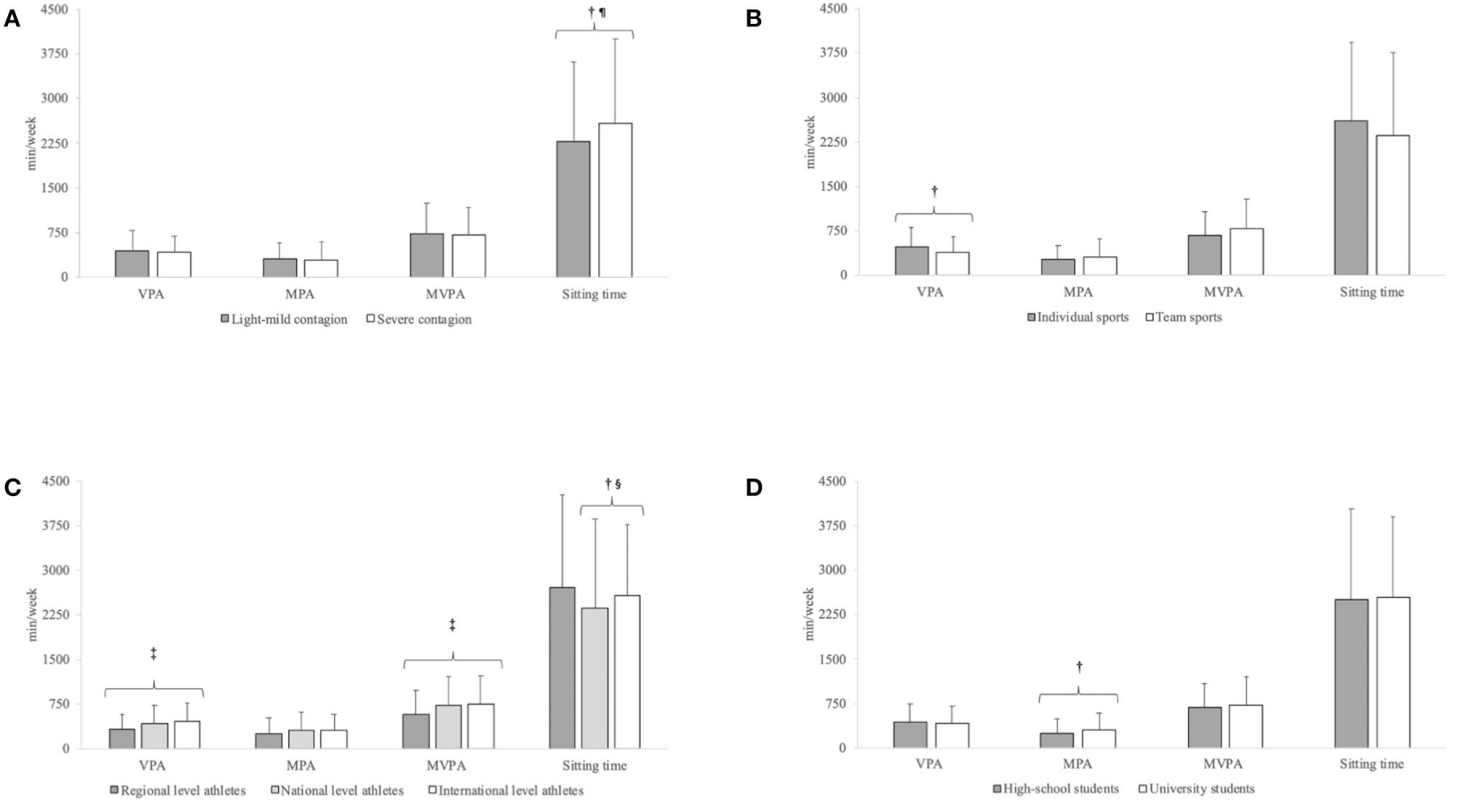
Weekly time of vigorous-intensity physical activity (VPA), moderate-intensity physical activity (MPA), and moderate to vigorous-intensity physical activity (MVPA) and sitting time in relation to the severity of contagion **(A)**, the typology of sport **(B)**, competition level **(C)**, and academic level **(D)**. †Significant difference between groups; ^¶^Groups differ only for weekend days sitting time; ^§^Groups differ for sitting time and weekdays sitting time; ^‡^Regional-level athletes significantly differ from both national- and international-level athletes.

According to the activity-level cutoffs of the guidelines for data processing and analysis of the IPAQ short and long forms (https://sites.google.com/site/theipaq/, 2020), the majority of the respondents (87.7%) met the international recommendations on physical activity for health (World Health Organization, 2010), reporting a high level of physical activity during the lockdown (81.8%); those who showed a low and a moderate level of physical activity were 12.3 and 5.9%, respectively. Figure 4 shows the weekly time of VPA and MPA, MVPA, and sitting in relation to the severity of contagion (Figure 4A), the typology of sport (Figure 4B), competition level (Figure 4C), academic level (Figure 4D). Overall, highest values emerged for sitting (*P* < 0.001), with the highest proportion of respondents declaring to sit <4 h per day (weekdays = 38.0%, weekend days = 82.5%) and the lowest proportion >8 h per day (weekdays = 28.4%, weekend days = 6.8%). Furthermore, a large amount of time sitting (*P* = 0.012), in particular during the weekdays (*P* = 0.026), was found for athletes competing at an international level with respect to national-level athletes and during the weekend day for athletes from countries with a severe contagion (*P* = 0.015). Despite no difference was found for PA level categories, university students performed more MPA (*P* = 0.010) with respect to their high school counterparts, athletes competing in individual sport performed more VPA (*P* = 0.014) with respect to team sports athletes, and regional level athletes engaged in less VPA and MVPA with respect to national (*P* = 0.01; *P* = 0.005, respectively)- and international (*P* < 0.001; *P* = 0.001, respectively)-level athletes.

The majority of the respondents declared to need about 3–4 weeks (40.0%) to recover their performance before the COVID-19 pandemic lockdown, followed by those declaring 1–2 weeks (32.3%) and more than 1 month (27.7%). Main effects emerged for sport typology (χ^2^ = 7.2, *P* = 0.03) and competition level (χ^2^ = 14.0, *P* = 0.007), with individual sport athletes in need of >1 month (*Z* = 2.7, *P* = 0.007) and regional-level athletes 1–2 weeks (*Z* = 3.2, *P* = 0.001), respectively.

## Discussion

The main results of the present study confirmed the hypothesis of the effects of COVID-19 in relation to the severity of the contagion, academic level, sport typology, and competition level on dual careers and active lifestyles of athletes. In particular, during the forced confinement, the student-athletes maintained an active lifestyle when compared to recommendations for the general population (World Health Organization, 2010), substantiating the relevant role of competitive sport participation in the maintenance of active lifestyles of the individuals and the need of cross-sectoral cooperation involving representatives of teachers, sports associations, and coaching experts for the enhancement of a lifelong health-oriented physical activity (European Commission, 2015). Finally, home training and e-learning were valuable resources during the lockdown, despite the decreased time dedicated to both studying and training.

### Student-Athletes' Commitment to Sport Before and During the COVID-19 Lockdown

At the elite level, several daily hours (~3 h) are devoted to training (Fiskerstrand and Seiler, 2004), with student-athletes reporting an average of ≥21 h week^−1^ spent in sport commitment (Condello et al., 2019). The present study not only substantiated the literature on the sport commitment of international student-athletes but also contributed to the quantification of the sport commitment of student-athletes competing at national (80.3% ≥11 h week^−1^) and regional (61.2% ≥11 h week^−1^) levels. Despite a difference in the sport commitment was expected in relation to the competition level of the athletes, the present data show that most athletes spend several hours per week to training and competition. In consideration of different national and/or sport-specific eligibility criteria for the student-athlete status, dual-career services might be not available for many talented and elite athletes who have to rely on their individual capability to arrange their academic and sport life (Capranica and Guidotti, 2016). Therefore, it is necessary that dual-career policies include athletes competing at national and regional levels to enable them to progress in both their academic and sport careers toward the highest outcomes they can achieve. In considering that athletic career is a multistage process (Stambulova and Samuel, 2020), this issue is particularly relevant for youth talented athletes aiming at a prospective elite sport career and in sport disciplines in which peak sport performances are expected in later years (Kalén et al., 2019; Solberg et al., 2019; Nikolaidis and Knechtle, 2020; Stambulova and Samuel, 2020).

Recently, particular concern has been expressed about the possibility that elite athletes can be exposed to some level of detraining during the lockdown, increasing the risk of injury and poor performance, upon restart (Eirale et al., 2020; Sarto et al., 2020). In the present study, both the quantity and the quality of athletes' training were affected as a result of the measures implemented to face the sudden outbreak of the COVID-19 pandemic. A large part of the respondents declared that they significantly changed training routines, lamenting the impossibility to access facilities and equipment as well as to train with teammates. As a consequence, they performed mostly conditioning training such as strength and aerobic trainings. Likely, the impossibility to perform sport-specific elements contributed to the reduction of training volume, intensity, and quality. Especially athletes from countries with severe anti-contagion lockdown measures and athletes competing in team sports considered the lack of training encompassing technical–tactical elements and the presence of teammates (Jukic et al., 2020) a problem.

During the COVID-19 lockdown, some coaches have provided home-based training programs and moral support especially to individual sports, competing at international level, and living in countries experiencing a severe contagion, which could mirror differences in economic opportunities and organization level of clubs and federations, peculiar requirements of sport disciplines, as well as restrictions to avoid the COVID-19 contagion. In considering that elite athletes are highly committed to sports showing high levels of mental toughness, motivation, positive energy, attitude control, self-confidence, negative energy control, visual imagery, determination to push their physical and mental limits (Coker-Cranney et al., 2018), the difference in this study between sports levels could be attributed to a significant motivation to sports of elite student-athletes (Gaston-Gayles, 2005; Guidotti et al., 2014; Lupo et al., 2015).

Another relevant aspect is the difference in the coach–athletes relationship between individual and team sports. According to the literature (Jowett and Ntoumanis, 2004; Jowett and Lavallee, 2007), the coach–athletes relationship implies a mutual and causal interconnection of their emotions, thoughts, and behaviors based on their closeness (i.e., the emotional attachment), commitment (i.e., their intention to maximize their athletic relationship), complementarity (i.e., their cooperative interactions), and co-orientation (i.e., empathic understanding) (Wachsmuth and Jowett, 2020). In particular, the findings of the present study substantiate the literature suggesting closer, more committed, and more complementary athlete–coach relationships in individual sports with respect to those of team sports (Rhind et al., 2012).

Several factors affect an athlete's condition, including psychological aspects, life events, and lifestyle behaviors. Definitely, the COVID-19 crisis has determined the lifestyle behavior of different communities (Sekulic et al., 2020; Xiang et al., 2020) mainly due to public movement restrictions forcing people to stay home and critical psychological repercussions due to dramatically disrupted daily routines and increased anxiety of contracting the virus. Recently, particular concern has been expressed about the active lifestyle of athletes outside of training (Sperlich and Holmberg, 2017; Izzicupo et al., 2019). Although the consequences of an inactive lifestyle in athletes' free time are not known, it may play a role, especially in particular moments of the competitive season (e.g., off-season, recovering from an injury). Therefore, it is important to try to understand whether athletes maintain an active lifestyle during the lockdown. This aspect could affect both the fitness and mental health of athletes. In the present study, despite during the lockdown almost the totality of the student-athletes reported a high level of physical activity with respect to that recommended for the general population (World Health Organization, 2010), they also declared a significant amount of time spent sitting, especially when living in countries experiencing a severe contagion. It is possible to speculate that these conditions resemble or even exacerbate off-training physical behaviors of athletes (Sperlich and Holmberg, 2017; Izzicupo et al., 2019). Studies on vascular, metabolic, and muscular adaptations to unloading (e.g., uninterrupted sitting and bed rest) as well as on post-injury conditions (Milsom et al., 2014) or the detrimental effects of the off-season (Suarez-Arrones et al., 2019) allow some speculations. Findings indicate that prolonged and uninterrupted sitting is associated with body fatness in highly trained athletes (Júdice et al., 2014); acute lower limbs dysfunction in healthy young subjects (Padilla and Fadel, 2017); increased sympathetic and renin–angiotensin system activity (Young and Leicht, 2011), plasma fibrinogen (Izzicupo et al., 2020), hematocrit, hemoglobin, and red blood cell count and a reduction in plasma volume (Howard et al., 2013); modified glucose and lipid metabolisms (Bergouignan et al., 2009; Stephens et al., 2011); changes in muscle size and architecture and tendon mechanical properties (De Boer et al., 2007; de Boer et al., 2008); muscle loss (Miles et al., 2005); and reductions in muscle strength, power, and rate of force development (Mujika and Padilla, 2001; De Boer et al., 2007; Bosquet et al., 2013; Rejc et al., 2018). While the actual effects of sitting behaviors observed during COVID-19 on the athletes' condition remain to be elucidated, the resumption of elite sports with congested competing calendars could put athletes at several health risks. Factually, scientific evidence associates a high risk of injury to a short preseason (Eliakim et al., 2018), a low number of training sessions during preseason (Ekstrand et al., 2020), and a lack of sport-specific training (Verrall, 2005). Therefore, proper recovery interventions (Tessitore et al., 2007) and monitoring strategies (McGuigan, 2017; Teixeira et al., 2018) are envisaged, with particular attention needed for athletes who might not have the same training possibilities of elite athletes. Finally, the extreme mutability and unpredictability of the COVID-19 contagion, incubation period, and clinical conditions urge maximal caution on the decision during the current and next sport seasons and events (Eirale et al., 2020; Toresdahl and Asif, 2020).

### Student-Athletes' Commitment to Academics Before and During the COVID-19 Lockdown

In line with the literature on elite student-athletes (Condello et al., 2019), before the lockdown, the majority of the respondents declared an academic commitment ≥21 h week^−1^ (38.9%) and 11–20 h week^−1^ (31.1%), with some difference in relation to the sport typology and academic levels. In particular, university students and international-level athletes declared to spend less time to academics with respect to their high school and to regional- and national-level counterparts, respectively. It is possible to affirm that the condensed training and competition schedules to compete at international level reduce significantly the time to be dedicated to academics. Furthermore, busy class schedules and compulsory attendances are more frequent at high school level with respect to university level.

During the lockdown, a general reduction in studying time was observed, despite student-athletes living in countries under a severe contagion being more involved in studying with respect of their counterparts living in countries experiencing light–mild contagion. Such a reduction seems counterintuitive when considering the increased availability of home time. However, it is possible to speculate that the unpreparedness of some institutions to promptly switch from face-to-face to online lessons, the limited availability of adequate IT equipment at home due to the massive demand of other siblings' e-learning and to parents in smart working, and limited fast Internet connection might have strongly affected equity in education (Burgess and Sievertsen, 2020). Furthermore, significant grief, stress, anxiety, frustration, and sadness for an athlete could be due to canceled training and competitions (Toresdahl and Asif, 2020). In considering that a study on the psychological health of college students conducted in China during the COVID-19 epidemic showed how social support was a protective factor against anxiety experienced during the pandemic (Cao et al., 2020), the support of significant figures, such as coaches and teachers, could have played a crucial role (Toresdahl and Asif, 2020). Actually, a strong detrimental impact on perceived stress and psychobiosocial states has been reported on Italian athletes living under severe COVID-19 restrictions (di Fronso et al., 2020). Coherently, student-athletes from countries under severe contagion perceived their teachers being less supportive with respect to that of those living in countries experiencing a light–mild contagion. It is possible to speculate that the abrupt and inflexible decision of Italian, Spanish, and Portuguese governments to close schools and universities increased the vulnerability of students and their need of support to cope with distance learning and digital skills, especially in these countries tied to traditional didactics.

In addition to their professional educational support, the teachers provided the students-athletes also moral and psychological support, perceived slightly less frequently with respect to that provided by the coaches. In considering the educative role of the coaches (Publications Office of the European Union, 2020), there is a need for a strict cooperation between teachers and coaches in helping dual-career athletes in pursuing both academic and sport paths. In fact, mainly international-level athletes reported specific dual-career support in terms of psychological assistance and tutoring. This aspect should not be surprising when considering that eligibility for dual-career programs is often exclusively including elite athletes (Capranica and Guidotti, 2016). The human right of education (The United Nations, 1948, art. 21.3) and the youth right to play (The United Nations, 1989) are both enshrined to guarantee the full development of the potentials of the individuals. Despite the experienced times of hardship might have accentuated inequalities, the COVID-19 crisis might also have allowed creative opportunities to build strong relationships and future alliances between dual-career service providers and academic and sport staff for providing coordinated support to student-athletes.

### Benefits of Dual Careers and Future Opportunities

In considering both the time dedicated to sports and academic commitments, the results from the present study are in line with the literature demonstrating that student-athletes dedicate relevant efforts to both careers. When unexpected life events break in, resilience is a crucial factor. In general, student-athletes possess this characteristic, being strongly committed to overcome difficulties in pursuing their dual career goals (Gaston-Gayles, 2005; Guidotti et al., 2014; Lupo et al., 2015) and being able to transfer the skills acquired from one field to another (Comeaux and Harrison, 2011; Aquilina, 2013; Sorkkila et al., 2017; Barger and Seward, 2018; Stambulova et al., 2020). Therefore, the majority of the student-athletes participating in this study perceived their status useful to cope with the COVID-19 emergency. When asked to compare themselves with their non-dual-career counterparts, most of the respondents answering to open-ended questions (36.9%) believe that pursuing dual careers is useful for learning effective time management (36.9%), finding higher motivation (24.3%), discipline (8.7%), and better health (2.9%), even combining some of those skills (17%). Time and life management, as well as motivation and discipline, has been extensively described in previous studies as characterizing student-athletes (Comeaux and Harrison, 2011; Aquilina, 2013; Barger and Seward, 2018). With respect to high school counterparts, the student-athletes enrolled at university tend to feel that their dual-career skills are more useful probably due to their more mature and independent status and a longer experience of dual careers in preparation for their future transition in the labor market.

However, almost 40% of student-athletes declared not to perceive a positive effect being dual-career athletes. This could be due to the lack of solid support structures for the dual-career athlete (Aquilina and Henry, 2019). The COVID-19 situation involved unexpected circumstances in which, depending on the dual-career and educational model in force in the Member States, each Member State set how the educational and sport systems should be adapted. It was the educational institution and sport body that made specific decisions, or even each teacher and coach were left free to make the adaptations considered appropriate (Abenza-Cano et al., 2020). This could have determined that some student-athletes were left unprotected, not feeling the support of the agents involved and seeing the practice of sport more as an obstacle than as a benefit. It is therefore necessary to design policies to support dual careers at all levels.

### Limitations, Future Lines of Research, and Implications

To the authors' knowledge, this is the first study on sport, education, and lifestyles of student-athletes, providing also their perception of dual-career benefits during the COVID-19 pandemic. However, this work presents some limitations. Despite a lack of validated surveys to collect relevant information on dual careers in these exceptional circumstances, the *ad hoc* created and validated questionnaire might have neglected to address many interesting aspects of confinement, such as mood, anxiety, and quantity and quality of sleep, which might affect the resilience of student-athletes during the COVID-19 pandemic lockdown. Despite the large sample of participants, the information mainly derived from six Member States (e.g., Italy, Romania, Spain, Latvia, Portugal, and Slovenia) with an uneven sample size, which urge some caution of data interpretation and limit the generalizability of the findings. Furthermore, the provisions relating to school, sport, and other aspects of daily life were implemented at different times and with different degrees of restriction in the Member States, ranging from severe constraints allowing to go out only for shopping food and medicines (e.g., Italy) to mild restrictions with free access to schools, restaurants, and malls (e.g., Latvia). To prevent heterogeneity of findings due to changes in the restrictions over time, the data collection was limited to 2 months.

Therefore, further studies allowing inferential statistics encompassing prediction formulas with regression analysis and probability of occurrence with odd ratio analysis are required to understand the needs of student-athletes in normal and exceptional situations. This information could allow identifying effective adaptations at both the educational and sporting levels and to optimize the symbiosis between these sectors. In addition, studies could be carried out to analyze the changes that a real support program for student-athletes could have on the determinants and dual-career success during both normal and exceptional circumstances. This would be especially important for athletes who have been shown to be more vulnerable due to a young age, severe restrictions, or lack of flexible dual-career programs or financial support. Research is also needed to investigate the long-term impact of the lockdown on sport performance and injury occurrence as a result of the change in the type and volume of training.

From a practical point of view, in highlighting a lack of dual-career support of youth athletes, the present findings call for a sound implementation of policies and services to allow the holistic development of the individual for achieving high-level sport performances and tertiary education. In particular, it is necessary for sports staff (e.g., coaches, physical trainers, managers) to be aware of proper adaptations when training and competitions are resumed after a period of decreased activity with confinement to avoid injuries and to restore physical condition and performance. This seems particularly necessary for the athletes who radically changed their training such as team sport players.

## Conclusions

Undoubtedly, the COVID-19 outbreak impacted worldwide both the educational and sports systems with relevant consequences for students and athletes. Notwithstanding, the current crisis could leave new opportunities behind. Although distance learning is not a new concept, so far, the education system has struggled to adopt this method of teaching. In a few months, the COVID-19 pandemic forced secondary and tertiary educational institutions to develop sustainable e-learning systems. In pre-pandemic times, online teaching suffered the adversity of teachers, which was an obstacle for the adoption of e-learning in dual-career support programs. Confronted with this emergency, teachers successfully attempted to do their best through online programs, social platforms, and educational systems. This legacy cannot be lost. Thus, e-learning and academic flexibility should be maintained to guarantee in the future adequate support to student-athletes. Furthermore, teams of sport and academic actors should cooperate to envisage and strengthen effective dual-career strategies to respond, care, and protect a future for student-athletes.

## Data Availability Statement

The raw data supporting the conclusions of this article will be made available by the authors, without undue reservation.

## Ethics Statement

The studies involving human participants were reviewed and approved by The Institutional Review Board of the University of Rome Foro Italico approved this study (CAR46/2020) carried out in agreement with the Declaration of Helsinki. The data were analyzed anonymously. Written informed consent to participate in this study was provided by the participants' legal guardian/next of kin.

## Author Contributions

PI and LC: conceptualization, design of the study, and formal analysis. PI, AD, and BG: methodology. PI, IA, UB, FC-A, MD, AF, JG-R, BG, AL-A, LM, AP, L-ER, C-MR, O-MR, HS, JS, RV-C, and VV: data collection. PI, BG, and LC: data curation. PI, AD, BG, and LC: writing—original draft preparation. IA, UB, AP, and JS: visualization. AD, AS-P, AF, and IA: supervision and editing. AP: funding acquisiti the manuscript.

## Conflict of Interest

The authors declare that the research was conducted in the absence of any commercial or financial relationships that could be construed as a potential conflict of interest.

## Supplementary Material

Supplementary File 1 Survey on the sport and academic commitments, support, and daily physical activity of student-athletes during the lockdown.

Supplementary File 2 Socio-demographic characteristics of the student-athlete population in the participating countries.
