# Supplementary material for: Dual Careers of Athletes During COVID-19 Lockdown
Source: Front Psychol. 2021 Apr 1;12:657671. doi: 10.3389/fpsyg.2021.657671 (PMC8047065; doi:10.3389/fpsyg.2021.657671)
Supplement: Supplementary File 1 — Survey on the sport and academic commitments, support, and daily physical activity of student-athletes during the lockdown. [file Data_Sheet_1.docx]

**Dual career of athletes during COVID-19 lockdown**

**Supporting information 1**

**Survey on the sport and academic commitments, support, and daily physical activity of Student/Athletes during the lockdown.**

**Socio-demographic items**

Q1) I am: (dichotomous: female, male)

Q2) Year of birth: (single response)

Q3) My Country is: (single response)

Q4) I am enrolled at university level: (single response: Bachelor, Master’s, PhD, High School, Other)

Q5) My major is: (drop-down list: Art-Art history-Music-Dance-Theatre, Biology-Biochemistry, Business-Economics-Administration, Communication-Multimedia-Journalism, Computer Sciences, Earth-Ocean-Atmospheric-Environmental Sciences, Education, Engineering-Bioengineering, Health, Sciences-Nutrition, History, Language-Foreign languages, Law, Liberal Arts, Literature, Mathematics, Medicine, Military Science, Naval Sciences, Pharmacy, Philosophy, Physics, Political-International Sciences, Psychology, Sociology-Social Sciences, Sport Sciences/Physical education, Statistics, Women-Gender-Sexuality Studies, Other)

Q6) Your sport is: (single response)

Q7) What is the level at which do you compete ? (single response: Regional, National, International)

**The athletes’ engagement in sport and university before and during the COVID-19 lockdown.**

Q8) In your country: in the last month the COVID-19 Pandemic: (single response: a lockdown of university only, the lockdown of sport, both the lockdown of university and sport)

Q9) In the last month before the lockdown due to the COVID-19 Pandemic: On average, how many hours per week were you actively engaged in elite sports (training, competition, physiotherapy etc.)? (drop-down list: 0-5, 6-10, 11-15, 16-20, 21-25, 26-30, 31-35, 36-40, 41-45, 46-50, 51-55, 56-60, >60)

Q10) During the first month of the lockdown due to the COVID-19 Pandemic: Did you engage in training? (dichotomous: yes, no)

Q11) If yes: On average, how many hours per week you actively engaged in training? (drop-down list: 0-5, 6-10, 11-15, 16-20, 21-25, 26-30, 31-35, 36-40, 41-45, 46-50, 51-55, 56-60, >60)

Q12) If yes: did you train at home? (dichotomous: yes, no

Q13) If yes: how was your training modified? (open answer)

Q14) If not: specify why (open answer)

Q15) In the last month before the lockdown due to the COVID-19 Pandemic: On average, how many hours per week were you actively engaged in academics (study, exams, laboratories, etc.)? (drop-down list: 0-5, 6-10, 11-15, 16-20, 21-25, 26-30, 31-35, 36-40, 41-45, 46-50, 51-55, 56-60, >60)

Q16) During the first month of the lockdown due to the COVID-19 Pandemic: On average, how many hours per week are you on average actively engaged in academics (study, exams, etc.)? (drop-down list: 0-5, 6-10, 11-15, 16-20, 21-25, 26-30, 31-35, 36-40, 41-45, 46-50, 51-55, 56-60, >60)

**Support and dual-career benefits**

Q17) During the first month of the lockdown due to the COVID-19 Pandemic: Did your coach support you? (dichotomous: yes, no)

Q18) If yes: What support your coach provided you? (open answer)

Q19) During the first month of the lockdown due to the COVID-19 Pandemic: Did your teachers support you? (dichotomous: yes, no)

Q.20) If yes: What support your teachers provided you? (open answer)

Q21) During the first month (dichotomous: yes, no)

Q22) If yes: What support did you receive as a student-athlete? (open answer)

Q23) In your opinion: Do you think that being a student-athlete helped you manage the COVID-19 Pandemic better than athletes not enrolled in higher education? (dichotomous: yes, no)

Q24) If yes: Why being a student-athlete helped you manage the COVID-19 Pandemic better than athletes not enrolled in higher education? (open answer)

Q25) If not: Why being a student-athlete did not help you manage the COVID-19 Pandemic better than athletes not enrolled in higher education? (open answer)

**Physical activity and sitting time**

Q26) Think about only those physical activities that you did for at least 10 minutes at a time. During the last 7 days, on how many days did you do vigorous physical activities like heavy lifting, fast walking? (drop-down list: 1, 2, 3, 4, 5, 6, 7, no vigorous activity - skip to question 26)

Q27) How much time did you usually spend on one of those days doing vigorous physical activities? (single response)

Q28) Think about only those physical activities that you did for at least 10 minutes at a time. During the last 7 days, on how many days did you do moderate activities like carrying light loads, sweeping, washing windows? (drop-down list: 1, 2, 3, 4, 5, 6, 7, no moderate activity - skip to question 30)

Q29) How much time did you usually spend on one of those days doing moderate physical activities? (single response)

Q30) During the last 7 days, how much time did you usually spend sitting on a weekday? (single response)

Q31) During the last 7 days, how much time did you usually spend sitting on a weekend day? (single response)

Q32) In your opinion: how much time you need to recover the sport performance you had before the COVID-19 Pandemic lockdown? (single response: 1-2 weeks, 3-4 weeks, more than 1 month)
