# Supplementary material for: Dual Careers of Athletes During COVID-19 Lockdown
Source: Front Psychol. 2021 Apr 1;12:657671. doi: 10.3389/fpsyg.2021.657671 (PMC8047065; doi:10.3389/fpsyg.2021.657671)
Supplement: Supplementary File 2 — Socio-demographic characteristics of the student-athlete population in the participating countries. [file Data_Sheet_2.docx]

**Dual career of athletes during COVID-19 lockdown**

**Supporting information 2**

**Socio-demographic characteristics of the student-athlete population in the participating countries.**

|  |  | **Italy** | **Romania** | **Spain** | **Latvia** | **Portugal** | **Other** |
| --- | --- | --- | --- | --- | --- | --- | --- |
| **Sex** | *Male (%)* | 56.1 | 56.9 | 50.0 | 87.5 | 60.7 | 60.0 |
|  | *Female (%)* | 43.9 | 43.1 | 50.0 | 12.5 | 39.3 | 50.0 |
| **Sport** | *Individual (%)* | 73.6 | 63.6 | 60.4 | 7.1 | 67.9 | 78.6 |
|  | *Team (%)* | 26.4 | 36.4 | 39.6 | 92.9 | 32.1 | 21.4 |
| **Level** | *International (%)* | 31.7 | 42.2 | 68.5 | 37.5 | 35.7 | 46.7 |
|  | *National (%)* | 49.4 | 28.1 | 25.9 | 43.8 | 50.0 | 50.0 |
|  | *Regional (%)* | 18.9 | 29.7 | 5.6 | 18.8 | 14.3 | 3.3 |
| **Study** | *High-school (%)* | 30.1 | 16.1 | 0.0 | 43.8 | 3.6 | 0.0 |
|  | *Bachelor (%)* | 56.1 | 66.1 | 83.3 | 46.9 | 42.8 | 70.0 |
|  | *Master’s (%)* | 13.8 | 17.8 | 16.7 | 9.4 | 53.6 | 30.0 |

Other are represented by Slovenian, Finnish, Kazakhstani, Croat, Serbian, and British student athletes.
